# Uptake of long chain fatty acids is regulated by dynamic interaction of FAT/CD36 with cholesterol/sphingolipid enriched microdomains (lipid rafts)

**DOI:** 10.1186/1471-2121-9-45

**Published:** 2008-08-13

**Authors:** Robert Ehehalt, Richard Sparla, Hasan Kulaksiz, Thomas Herrmann, Joachim Füllekrug, Wolfgang Stremmel

**Affiliations:** 1 Department of Gastroenterology, University Hospital Heidelberg, INF 410, 69120 Heidelberg, Germany

## Abstract

**Background:**

Mechanisms of long chain fatty acid uptake across the plasma membrane are important targets in treatment of many human diseases like obesity or hepatic steatosis. Long chain fatty acid translocation is achieved by a concert of co-existing mechanisms. These lipids can passively diffuse, but certain membrane proteins can also accelerate the transport. However, we now can provide further evidence that not only proteins but also lipid microdomains play an important part in the regulation of the facilitated uptake process.

**Methods:**

Dynamic association of FAT/CD36 a candidate fatty acid transporter with lipid rafts was analysed by isolation of detergent resistant membranes (DRMs) and by clustering of lipid rafts with antibodies on living cells. Lipid raft integrity was modulated by cholesterol depletion using methyl-β-cyclodextrin and sphingolipid depletion using myriocin and sphingomyelinase. Functional analyses were performed using an [3H]-oleate uptake assay.

**Results:**

Overexpression of FAT/CD36 and FATP4 increased long chain fatty acid uptake. The uptake of long chain fatty acids was cholesterol and sphingolipid dependent. Floating experiments showed that there are two pools of FAT/CD36, one found in DRMs and another outside of these domains. FAT/CD36 co-localized with the lipid raft marker PLAP in antibody-clustered domains at the plasma membrane and segregated away from the non-raft marker GFP-TMD. Antibody cross-linking increased DRM association of FAT/CD36 and accelerated the overall fatty acid uptake in a cholesterol dependent manner. Another candidate transporter, FATP4, was neither present in DRMs nor co-localized with FAT/CD36 at the plasma membrane.

**Conclusion:**

Our observations suggest the existence of two pools of FAT/CD36 within cellular membranes. As increased raft association of FAT/CD36 leads to an increased fatty acid uptake, dynamic association of FAT/CD36 with lipid rafts might regulate the process. There is no direct interaction of FATP4 with lipid rafts or raft associated FAT/CD36. Thus, lipid rafts have to be considered as targets for the treatment of lipid disorders.

## Background

Uptake of long chain fatty acids (LCFAs) is important for many cellular functions and the understanding of the uptake mechanisms is an important target for treatment of lipid disorders [1-4]. The molecular mechanisms of fatty acid transport across the plasma membrane are still a matter of debate and the predominating mechanism likely differs from cell to cell (for reviews see [5-8]). In general, two possible groups of mechanisms are discussed: *simple diffusion *and *saturable transport processes*. Whereas the uptake based on the passive diffusion process depends on the activity of intracellular metabolism creating a transmembrane downhill concentration gradient; the saturable process is regulated by expression of certain proteins and lipids at the plasma membrane. Much effort has been spent to identify candidate proteins that are directly involved in the facilitated fatty acid uptake mechanism. So far four candidates (FABPpm, FAT/CD36, FATP family proteins, caveolin-1) have been discussed [9-11]. Whereas FAT/CD36 and FABPpm are membrane associated fatty acid binding proteins that are thought to mediate their dissociation from albumin and accumulation at the outer leaflet of the plasma membrane [12], followed by flip-flop across the phospholipid bilayer to the cytosolic site; it has been suggested that FATPs are real transporters directly involved in the uptake process of LCFAs across the membrane bilayer [5]. However, recent data from our laboratory suggest that this group of proteins are rather enzymes that indirectly facilitate the translocation process by encompassing acyl-coA activity [13].

The composition of membrane lipids also modulates fatty acid uptake. In particular, there is increasing evidence that cholesterol is of crucial importance. Cholesterol depletion in 3T3 adipocytes, HMEC, HEK293 or HepG2 cells decreased LCFA uptake and this effect was reversible after re-addition of cholesterol [14-17]. Caveolin-1 has been suggested to regulate the cholesterol content of the plasma membrane [8,18] and LCFA uptake. Caveolin-1 can bind LCFAs [10]. Caveolin-1 knock out mice showed a reduced mass of adipocytes and increased serum free fatty acids, indicating that LCFA uptake into adipocytes might be impaired [19]. LCFA uptake is increased by caveolin-1 overexpression and inhibited in caveolin-1 knockout mouse fibroblasts [20] or by expression of the dominant negative caveolin-1 mutant CAV^DGV ^[16]. Interestingly, the inhibitory effects of CAV^DGV ^can be reversed by replenishing the cell membranes with cholesterol and can be mimicked by methyl-β cyclodextrin treatment [21].

All these studies point out that cholesterol is critically involved in LCFA uptake. However, little is known about the mechanisms by which cholesterol modulates this process. We have previously hypothesized that the association of FAT/CD36 with lipid rafts might determine this process [8]. Rafts are lateral assemblies of sphingolipids and cholesterol within cellular membranes involved in compartmentalization of membrane processes [22]. Biochemically, lipid raft constituents are characterized by their insolubility in low concentration of detergents such as Triton X-100 [23]. In this regard, it has been demonstrated that a fraction of FAT/CD36 associates with detergent-resistant membranes (DRMs) in a cholesterol-dependent manner [16]. The reduction of overall LCFAs uptake by cholesterol depletion was as effective as the specific inhibition of the FAT/CD36 function by sulfo-*N*-succinimidyl oleate (SSO). Simultaneous treatments had no additional effect, suggesting that both procedures target the same cellular compartment [16].

The most straightforward interpretation of these findings is that there are two pools of FAT/CD36 at the plasma membrane, one associated with lipid rafts, in which LCFAs are transported, and another outside of rafts, where no transport occurs. That means, FAT/CD36 has to reside in lipid rafts to facilitate FA uptake. Cholesterol depletion would shift FAT/CD36 into the surrounding lipid bilayer and make this protein non-functional. If FAT/CD36 facilitated uptake of fatty acids requires that the receptor is associated with lipid rafts, then regulation of this association may represent a mechanism by which cellular uptake of fatty acids can be regulated. In this paper we provide evidence demonstrating that association of FAT/CD36 with lipid rafts is critical for the uptake process to occur.

## Methods

### Reagents and antibodies

Methyl-β-cyclodextrin, sphingomyelinase and myriocin were from Sigma. Antibodies used were mouse anti-placenta alkaline phosphatase (PLAP) (Dako Cytomation), rabbit anti-PLAP [24]; rabbit anti-FATP4 (C4 anti FATP4 described in [13]); mouse anti-FLAG (Sigma), Cy5 and Cy3 donkey anti-mouse/rabbit (Jackson Immuno Research); mouse monoclonal antibody anti GFP 3E6 (Invitrogen); rabbit anti GFP KG77 [25]; mouse anti-FAT/CD36 (Biosource).

### Constructs

GFP-FATP4 have been described previously [13]. CD36-FLAG was kindly provided by Douglas M. Lubin, Washington University School of Medicine, St. Louis, MO. GFP-TMD is a membrane anchored version of the green fluorescent protein. The signal sequence of human CD8 (MALPVTALLLPLALLLHAARP) is followed by an epitope tag (VSV-G; YTDIEMNRLGK). Next is EGFP, followed by a 16 amino acid glycosylation tag from human rhodopsin (NGTEGPNFYVPFSNAT) and the transmembrane domain of podocalyxin (EFEDRFSMPLIITIVCMASFLLLVAALYGCCHRK). This plasmid is identical to the construct GFP-tail described in [26] except that the cytoplasmic tail has been removed and behaves like a non-raft protein [26].

### Oleate uptake

Cumulative uptake of oleate was based on Stremmel and Berk [27]. Adherent cells were incubated for 5 min at 37°C with [3H]-oleate solution 170 μM (0.68 μCi/ml [3H]-oleate (Amersham)) BSA fatty acid free (Sigma) in PBS; After stopping and washing with ice-cold 0.5% BSA in PBS, cells were lysed with 1 M NaOH and aliquots analyzed for protein concentration (Biorad) and radioactivity by scintillation counting as before [13].

### Cells, transient transfection, cholesterol/sphingolipid depletion

COSJ (ATCC CRL-1651) and Vero (CCL-81) cells were maintained under standard tissue culture conditions with the appropriate culture media (COSJ: D-MEM Invitrogen 4,5 g glucose/L 10% FBS 2 mM L-Glutamine and Vero: D-MEM Invitrogen 1 g glucose/L 5% FBS 2 mM L-Glutamine). Cells grown to near confluency (10 cm^2^) were transfected with 4 μg plasmid-DNA and 10 μl lipofectamine 2000 (Invitrogen). Analysis was performed 16–20 h hours after transfection. For cholesterol depletion the cells were treated for 30 min with 10 mM methyl-β-cyclodextrin (MβCD) in DMEM. Cholesterol determinations were done using the Amplex Red Cholesterol Assay kit (Molecular Probes). For inhibition of sphingolipid synthesis the cells were seeded at a density of 0.2 × 10^6 ^in 10 cm^2 ^dishes and were grown for 3 d in complete medium in the presence of 5 μM myriocin. During this period the medium was changed once. Growing COS cells in the presence of myriocin for a longer time caused the cells to detach and undergo apoptosis. The extent of sphingolipid-depletion was estimated microscopically on cells incubated for 30 min with 25 μg/ml of rhodamine-conjugated cholera toxin subunit B (Rh-CTB) (Molecular Probes) at 4°C, which was followed by a wash and subsequent fixation in 4% paraformaldehyde in PBS (PFA). The expression of FATP4 and FAT/CD36 for the different conditions were in initial experiments by Western blotting and found not to differ significantly (data not shown).

### Immunofluorescence and antibody-induced patching

For immunofluorescence microscopy, cells were fixed for 4 min with 4% PFA at 8°C followed by an incubation in methanol at -20°C for 4 min. Fixed cells were incubated for 1 h at room temperature with the appropriate dilution of antibodies in PBS/0.2% gelatine (see below). After three washes with PBS/0.2% gelatine they were incubated with the respective secondary antibodies in PBS/0.2% gelatine for 1 h at room temperature.

To aggregate raft proteins the respective antibodies were diluted in CO_2 _independent medium (GIBCO) containing 2 mg/ml BSA. The polyclonal antibodies against PLAP were diluted 1:35; the monoclonal anti-GFP (3E6) and anti-FAT/CD36 1:50. The cells were incubated for 45 min with the respective combination of antibodies at 10°C, briefly washed and further incubated for 45 min at 10°C with mixed fluorescently labelled secondary antibodies. Cy3-labelled secondary antibodies were diluted 1:500, and the Cy5-labelled ones 1:100. The cells were fixed as described above. Fluorescent images were acquired on an Olympus microscope and arranged with Adobe Photoshop.

### Preparation of detergent resistant membranes (DRMs)

Detergent extraction with Triton X-100 was performed as described before for N2a cells [25]. Cells were grown in 3.5 cm dishes, transfected and 10–12 h later washed once with PBS and scraped on ice into 1.5 ml homogenisation buffer (250 mM Sucrose, 10 mM Hepes, 2 mM EDTA) and after centrifugation (5 min 2000 rpm) cell pellets were homogenized in homogenisation buffer containing 20 μg/ml each of chymostatin, leupeptin, antipain and pepstatin A (Sigma) through a 26 G needle and centrifuged for 5 min at 3000 rpm. The postnuclear supernatant was subjected to extraction for 30 min at 4°C in 1% Triton X-100. The extracts were adjusted to 40% OptiPrep (Axis-Shield) and overlaid in a TLS 55 centrifugation tube with 30% OptiPrep/TNE, and TNE (25 mM Tris-HCl, pH 7.4, 150 mM NaCl, 2 mM EDTA old protocol/25 mM Tris-HCl, pH 10.8, 150 mM NaCl, 5 mM EDTA new protocol since 03.2007). The gradients were centrifuged at 400000 *g *in a Beckman SW41 rotor for 20 h at 4°C. Fractions were obtained and used for Western blotting as described [16] and protein levels quantified by densitometry.

### Statistical analysis

All values are reported as mean and standard error of the mean (SEM). The Kruskal-Wallis test was used to test for statistical significance. Probability values of p < 0.05 were set as threshold for statistical significance.

## Results

### Cholesterol depletion inhibits long chain fatty acid uptake in COS cells

**Figure 1:**
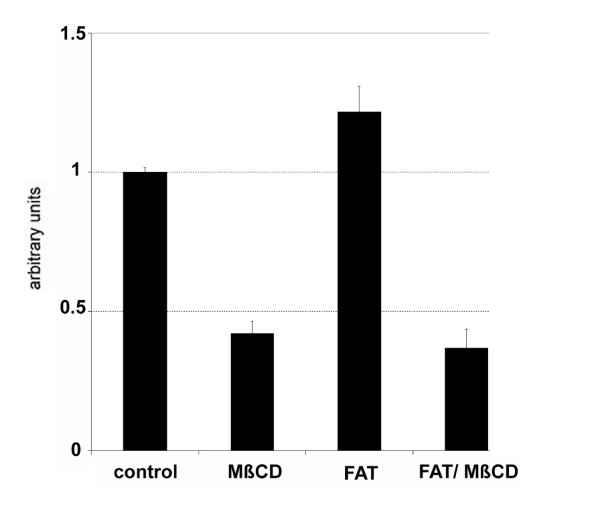
**Cholesterol depletion inhibits [3H]-oleate uptake.** COS cells were transiently transfected with FAT/CD36 and treated with 10 mM MβCD leading to a ~50% decrease in total cellular cholesterol (not shown). Afterwards [3H]-oleate uptake within 5 min was analysed. FAT/CD36 increased [3H]-oleate uptake significantly (p < 0.05). Cholesterol depletion decreased overall fatty acid uptake to less then 50% of control cells (p < 0.05). Data are expressed as mean and SEM of at least n = 6 experiments. The ratio has been arbitrarily set to 100% in cells that were not cholesterol depleted.

To study the role of lipid rafts in long chain fatty acid uptake we first analyzed again the effect of cholesterol depletion also in COS cells. Cholesterol depletion was done by treatment with the complexing agent methyl-β-cyclodextrin (MβCD) that extracts plasma membrane cholesterol. Immediately prior to testing uptake of [3H]-oleic acid, COS cells were treated with 10 mM MβCD for 30 min. By this the cholesterol levels could be reduced to 50% of those of control cells. To easily monitor the role of FAT/CD36 in fatty acid uptake cells were transiently tranfected with cDNA of FAT/CD36. Using lipofectamine as a mediator up to 50% of cells could be transfected. Overexpression of FAT/CD36 resulted in an increased overall uptake of [3H]-oleic acid by >20% within 5 min (Figure 1). Depletion of cholesterol with MβCD decreased fatty acid uptake by >50% in both control cells and FAT/CD36 transfected cells (Figure 1). These results show again that cholesterol is critically involved in [3H]-oleate uptake.

### Inhibition of sphingolipid synthesis decreases LCFA uptake

Rafts are cholesterol and sphingolipid-enriched microdomains. To find out whether sphingolipids are also important for LCFA uptake, we tested the effect of inhibition of sphingolipid biosynthesis.

**Figure 2:**
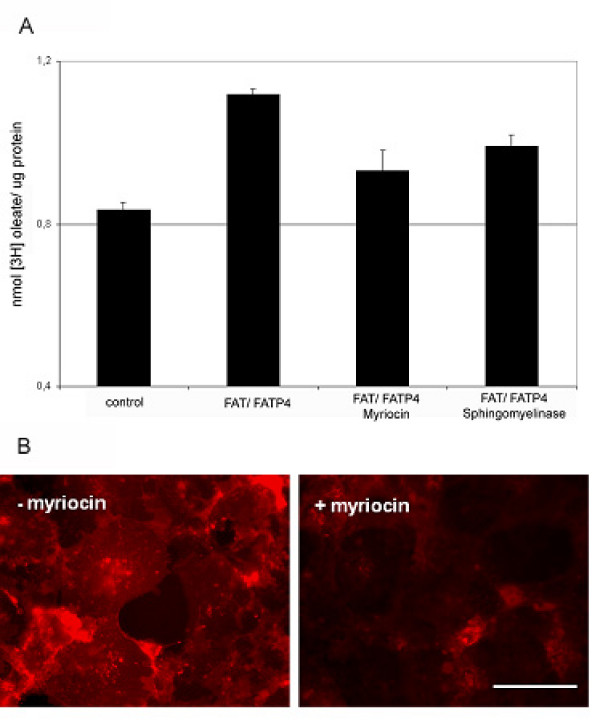
**Myriocin and sphingomyelinase treatment decreases [3H]-oleate uptake.** COS cells were either grown before cotransfection of FAT/CD36 and FATP4 for 3 d in the presence or absence (control) of 5 μM myriocin or were treated after co-transfection for 15 min with 1 U sphingomyelinase. (A) Overexpressing FAT and FATP4 increased [3H]-oleate uptake significantly (p < 0.05). Myriocin and sphingomyelinase treatment inhibited [3H]-oleate uptake significantly (p < 0.05). Data are expressed as mean and SEM from n = 3 independent experiments. (B) Staining of COS cells with Rh-CTB after 3 d treatment with 5 μM myriocin. The amount of the ganglioside GM1 on the surface is reduced after myriocin treatment. Bar: 200 μm. Both images in panel B were recorded with the same exposure time.

COS cells were cultured in the presence of 5 μM myriocin, which inhibits the first step of de novo ceramide synthesis by interacting with the serine-palmitoyl transferase or treated with 1 U of sphingomyelinase that is supposed to deplete plasma membrane sphingolipids. Cells were either treated for 3 d with myriocin before cotransfection of FAT/CD36 and FATP4 or treated 1 d after cotransfection for 15 min with 1 U of sphingomyelinase. [3H]-oleate uptake within 3 min was measured. Both treatments resulted in a reduction of [3H]-oleate uptake by 17 ± 3% (myriocin) and 15 ± 2% (sphingomyelinase), respectively (Figure 2A). The ganglioside GM1 localizes to lipid rafts and its plasma membrane level is altered by inhibition of ceramide synthesis [28]. We monitored the effect of myriocin and sphingomyelinase by staining of the ganglioside GM1 with rhodamine-conjugated cholera toxin subunit B (Rh-CTB). Myriocin treatment led to a decrease in staining, suggesting a decreased amount of total sphingolipids (Figure 2B). Sphingomyelinase resulted in similar results (data not shown). Thus, [3H]-oleate uptake can also be altered by depletion of sphingolipids.

### FAT/CD36 co-patches with placental alkaline phosphatase and segregates from non-raft marker GFP-TMD

**Figure 3:**
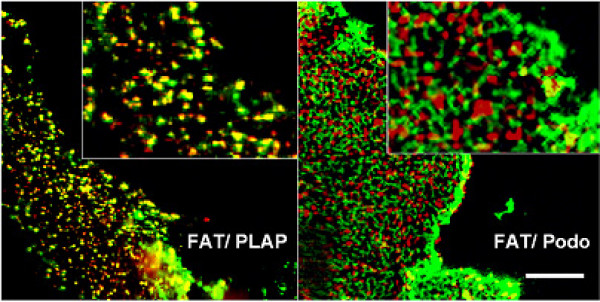
**Co-patching of PLAP and GFP-TMD (Podo) with FAT/CD36.** 20 h after transient transfection, Vero cells were incubated for 45 min at 10°C with respective primary antibodies, washed and incubated for 45 min with mixed Cy5 and Cy3 fluorescently-labelled secondary antibodies. PLAP and GFP-TMD were patched with polyclonal rabbit anti PLAP and polyclonal serum KG77 against GFP, respectively. Patching of FAT/CD36 was achieved with monoclonal antibody from Biosource international. FAT/CD36 co-localized with the raft marker protein PLAP and segregated from GFP-TMD. Bar: 10 μm.

**Figure 4:**
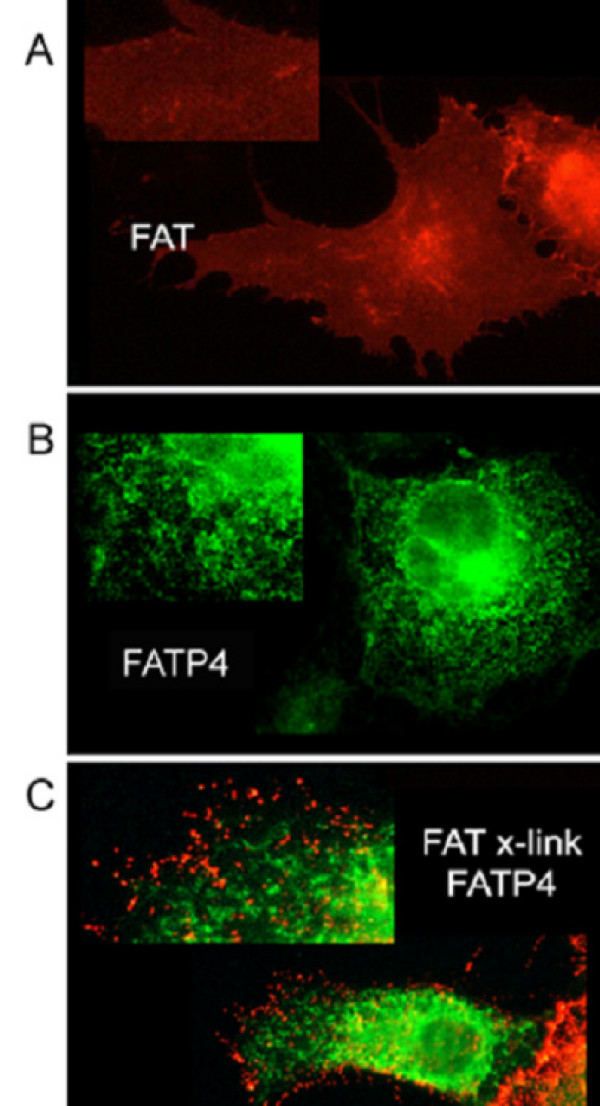
**FAT/CD36 does not co-localize with FATP4.** 20 h after transient transfection with FAT/CD36 and FATP4-GFP, FAT/CD36 was clustered with anti-FAT/CD36 antibody from Biosource and Cy3 fluorescently labelled secondary antibody. (A) FAT/CD36 is mainly localized at the plasma membrane. (B) FATP4-GFP shows a reticular staining pattern as described before [13] representing ER membranes. (C) Patched FAT/CD36 does not show any co-localisation with FATP4-GFP.

Lipid rafts are small and highly dispersed at the plasma membrane. In fibroblasts using photonic force microscopy it has been found that individual rafts have a size of about 50 nm and contain approximately 3000 sphingolipid molecules and probably a subset of 10–30 protein molecules [29]. Therefore they are too small to be resolved by light microscopy. However, raft and non-raft markers can be cross-linked with antibodies into visible patches [25,30,31]. Raft proteins co-patch with each other and segregate away from non-raft markers. Thus, cross-linking with antibodies can be used as a tool to analyse raft association. We therefore tested whether antibody cross-linking induces co-patching of FAT/CD36 with a raft marker, the glycosyl phosphatidylinositol (GPI)-anchored protein placental alkaline phosphatase (PLAP). As a non-raft marker, we used GFP-TMD. COS cells were transfected and the respective plasma membrane antigens cross-linked with antibodies recognizing the extracellular part of the membrane proteins. FAT/CD36 clearly co-localized together with PLAP at the plasma membrane in the majority of cells and segregated from GFP-TMD (Figure 3). As expected [13] FATP4 showed an intracellular pattern completely different from FAT/CD36. As it could not be detected at the plasma membrane no co-patching with FAT/CD36 (Figure 4) could be seen. This supports again our previous data [13] that FATP4 is an enzyme at the ER level, indirectly involved in fatty acid uptake due to enzyme activity.

### Antibody induced cross-linking increases the association of FAT/CD36 to detergent resistant membranes

Various proteins associate to lipid rafts with different kinetics and partition coefficients [32]. It has been shown that antibody-induced patching may stabilize association of raft proteins with detergent-resistant membranes (DRMs) [22,31]. We therefore analysed the association of FAT/CD36 to DRMs under cross-linking with antibodies.

**Figure 5:**
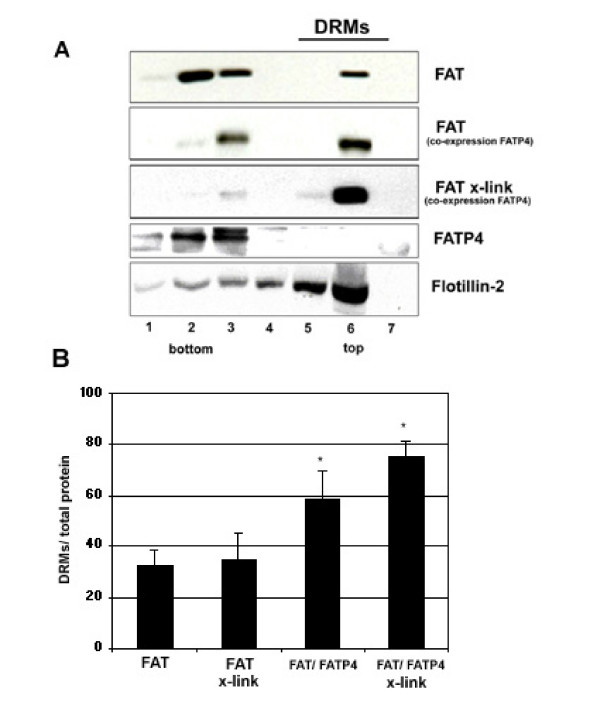
**Effect of antibody cross-linking on association of FAT/CD36 to DRMs.** 20 h after transient transfection of FAT/CD36, FATP4 or both COS cells were were lysed in 1% Triton X-100/TNE at 4°C. (A) After floatation in an OptiPrep step-gradient FAT/CD36 was found in two pools, in DRMs and in soluble membranes (lane 1–3). Co-expression of FATP4 resulted in an increased relative amount of FAT/CD36 found in DRMs (lane 6). Antibody cross-linking of FAT/CD36 using an mouse anti human FAT/CD36 antibody from Biosource shifted FAT/CD36 towards the DRM fractions (lane 6). No significant amount of FATP4 was found in DRMs, indicating that FAT/CD36 and FATP4 might be in distinct compartments within the cell. Flotillin-2, a typical raft protein was used as a control to estimate the quality of DRM isolation. The results are representative of three others experiments carried out independently. (B) Quantification; FATP4 expression and antibody-induced patching significantly increased the amount of FAT/CD36 in the top two fractions (DRM associated). The amount in the top two fractions was correlated to the total amount of protein in all fractions. Data are expressed as mean and SEM of n = 3 experiments. Asterisk indicates significant differences to cells transfected with FAT/CD36 only (p < 0.05).

In initial experiments COS cells were transfected with FAT/CD36 and then extracted with Triton X-100 and subjected to OptiPrep™ step gradient centrifugation. Under those conditions in three independent experiments no significant effect of antibody cross-linking could be detected. FAT/CD36 was found in both Triton X-100 resistant and soluble fractions showing that there are two pools of FAT/CD36 in cellular membranes. However, when FATP4 was co-expressed with FAT/CD36 raft association of the FAT/CD36 was increased (Figure 5). Co-expression of the cytosolic green fluorescent protein (GFP) or the ER marker sec61-GFP [33] did not change DRM association of FAT/CD36 (data not shown). Performing cross-linking under co-expressing conditions revealed a clearly increased fraction of FAT/CD36 in the DRM fraction (Figure 5). Therefore, DRM association of FAT/CD36 can be increased by cross-linking with antibodies, which probably reflects increased raft affinity caused by oligomerization. Similar results have been demonstrated for other proteins, which by forming oligomers increase their raft association [34-39]. The fact that increased association of FAT/CD36 to DRMs occurs especially in FATP4 overexpressing cells indicates a promoting effect of FATP4 for FAT/CD36 raft association.

### Cross-linking of FAT/CD36 with antibodies increases oleate uptake

**Figure 6:**
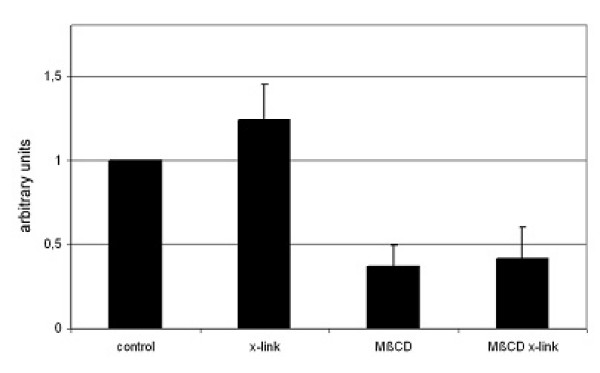
**Effects of antibody cross-linking (x-link) and cholesterol depletion on [3H]-oleate uptake.** Cells were transiently transfected with FLAG-FAT/CD36 and FATP4 and then treated or not for 30 min with 10 mM methyl-β-cyclodextrin (MβCD). Afterwards overall [3H]-oleate uptake within the first 5 min in presence or absence of anti-FAT/CD36 from Biosource was analysed. Quantification of three independent experiments is shown. The ratio has been arbitrarily set to 100% in cells neither cross-linked nor cholesterol depleted. Cross-linking increased overall fatty acid uptake significantly (p < 0.05). However under conditions of cholesterol depletion, antibody enhanced fatty acid uptake was not apparent.

If long chain fatty acid uptake were to take place in lipid rafts, then antibody cross-linking should not only induce co-patching with raft markers at the surface of living cells and increase association between FAT/CD36 and DRMs. It should also increase LCFA uptake, provided that the antibody does not neutralize LCFA binding or transport To find this out, we analyzed the effect of antibody cross-linking on overall [3H]-oleate uptake. Cells were co-transfected with FATP4 and FAT/CD36 and [3H]-oleate uptake analyzed within 5 min in the presence of anti-FAT/CD36 from Biosource (Figure 6). Antibody enhanced cross-linking indeed increased [3H]-oleate uptake significantly. We next examined the effect of cholesterol depletion on antibody-induced [3H]-oleate uptake. Immediately prior to the uptake assay transfected cells were treated for 30 min with 10 mM methyl-β-cyclodextrin and again uptake within 5 min was analyzed. After cholesterol depletion with methyl-β-cyclodextrin, [3H]-oleate uptake was no longer enhanced by antibody cross-linking (Figure 6). Thus, under conditions in which rafts are supposed to be disrupted (cholesterol depletion) increased uptake of [3H]-oleate due to cross-linking with antibodies is not detectable. Thus, lipid raft integrity is important for FAT/CD36 function in fatty acid uptake.

## Discussion

The data presented here strengthen the evidence that FAT/CD36 partition into lipid rafts and that LCFA uptake mediated by FAT/CD36 depends on this association.

First of all, we could show that LCFA uptake was critically dependent on the integrity of lipid rafts. Lipid rafts are cholesterol and sphingolipid enriched microdomains. Removal of raft lipids from cells leads to disruption of raft functions [22]. Consistent with previous results on other cells [14-17] we could demonstrate that by decreasing cellular levels of cholesterol, LCFA uptake in COS cells was also inhibited by > 50%. Additionally, we could show that by decreasing ceramide synthesis with myriocin or depleting membrane sphingolipids using external sphingomyelinase we were able to lower LCFA uptake. Sphingolipid depletion was less dramatic, probably because it is more difficult to lower cellular sphingolipid levels than cholesterol levels. The effects of sphingolipid synthesis inhibition may also be more pleiotropic because there are many different kinds of sphingolipids (sphingomyelins and glycosphingolipids). Nevertheless, we found that plasma membrane GM1 levels were reduced after myriocin treatment, demonstrating that not only cholesterol but also sphingolipids modulate LCFA uptake.

Another important finding supporting raft association was that after cross-linking with antibodies FAT/CD36 co-patched with placental alkaline phosphatase (PLAP), a GPI-anchored raft protein, in living cells and segregated from patches formed by cross-linking of GFP-TMD that served as a non-raft marker. This assay has previously been used to monitor if proteins associate with lipid rafts at the cell [25,39]. Antibody cross-linking of surface proteins leads to the formation of plasma membrane clusters that can be easily observed during light microscopy. It has been shown that co-patching is dependent on plasma membrane cholesterol, as patching is inhibited by cholesterol removal [31].

Antibody cross-linking increased DRM association of FAT/CD36 as was previously demonstrated for other raft proteins [25,31]. Because a considerable amount of FAT/CD36 was also found in the soluble fraction after detergent treatment, FAT/CD36 is probably found (in steady state) in two membrane pools, one raft associated and another localized in the surrounding bilayer. How partitioning between these two pools is regulated is an open question, but interestingly it has been shown that FAT/CD36 can dimerize [36]. Oligomerization of raft components is known to lead to increased raft affinity [37,38]. Therefore this mechanism might be important to regulate raft association of FAT/CD36.

It has been previously hypothesized that the detergent soluble fraction might represent intracellular FAT/CD36 and that upon cholesterol depletion FAT/CD36 might be stuck in the secretory pathway to the plasma membrane [16]. It has been described, that FAT/CD36 can indeed translocate between the plasma membrane and intracellular compartments [40]. However, recent data from Covey et al [17] using immunofluorescence and flow cytometry convincingly show that in 3T3-L1 adipocytes cholesterol depletion inhibits uptake of LCFAs without affecting FAT/CD36 or caveolin-1 distribution within the cells, indicating that cholesterol levels regulate LFCA uptake via a pathway that does not involve altered surface localization of FAT/CD36. Our cross-linking data, which were obtained on living cells at 10°C, can explain these findings because under these conditions intracellular antigens are not accessible to antibodies. Therefore increased DRM association of FAT/CD36 (Figure 5) after cross-linking with antibodies likely represent a horizontal recruitment of plasma membrane non-raft associated FAT/CD36 into raft domains. The enrichment of DRM associated FAT/CD36 could only be detected in presence of co-expressed FATP4. Whether the FATP4 acitivity as acyl-CoA synthetase drives cellular fatty acid influx and, thus, the requirement for raft associated FAT/CD36 or whether FATP4 is actively involved in the raft constitution process remains to be elucidated. FATP4 has been originally described to by a major fatty acid transporter at the apical membrane of enterocytes [11] and it has been speculated that both proteins might cooperate at the plasma membrane in fatty acid uptake [5,41]. However recent data could not support this view [13]. FATP4 seems rather to be an enzyme for acyl-CoA activity at the ER level and therefore indirectly increases fatty acid uptake. Deletion of FATP4 resulted in a perinatal lethality with a phenotype reminiscent of lethal restrictive dermopathy [42]. By histology the skin was characterized by a hyperproliferative hyperkeratosis with a disturbed epidermal barrier. Lipid analysis of the skin revealed an increased proportion of ceramides and cholesterol. Within the ceramide fraction very long chain fatty acid substitutes were significantly reduced [42]. These finding suggests that FATP4 is involved in ceramide biosynthesis, possibly by providing CoA activity for very long chain fatty acids. It is intriguing to speculate that this protein might therefore have also impact on lipid raft integrity. Rafts are enriched in sphingolipids (that are made from ceramides) and their long chain fatty acid substitutes have been implicated to be important to connect the outer and the inner layer of the microdomain. FATP4 would therefore be indirectly involved in the fatty acid uptake process by providing the molecules for a functional lipid raft assembly. This could explain our results of an enhanced raft association of FAT/CD36 after overexpression of FATP4.

In general clustering of lipid rafts can be achieved by different mechanisms [22]. Besides antibodies also other ligands (e.g. insulin), lectins or linker proteins have been discussed. Which would be a reasonable mechanism for FAT/CD36 clustering? It is conceivable that under physiological conditions the clustering of FAT/CD36 is mediated by binding of long chain fatty acids. It was indeed shown that the artificial, long chain fatty acid [^3^H]-sulfo-N-succinimidyl oleate (SSO) – when incubated with adipocytes – binds to FAT/CD36 with high affinity and is almost exclusively found in DRMs [16]. Thus the concentration of fatty acids presented to the plasma membrane may regulate the rate of fatty acid uptake by providing a sufficient functional platform in the form of FAT/CD36-raft complexes.

## Conclusion

Our data support a crucial role for lipid rafts in LCFA uptake. Compartmentalization of FAT/CD36 at the cell surface by lipid rafts seems to be important in regulating its involvement in LCFA uptake. Taken all data together it seems possible that fatty acid binding increases raft affinity of FAT/CD36. The protein is shifted from the surrounding bilayer to these microdomains were LCFA uptake is likely to occur.

## Abbreviations

LCFA: long chain fatty acids; SSO: sulfo-*N*-succinimidyl oleate; DRM: detergent resistant membrane.

## Authors' contributions

RE precipitated in the design of the study and carried out the immunofluorescent experiments. He drafted the manuscript. RS carried out the uptake and the floatation experiments. JF, WS, HK and TH participitated in the design of the study and helped to draft the manuscript. All authors read and approved the final manuscript.

## Achnowledgements

Work was supported by Stiftung Nephrologie and the Dietmar-Hopp-Stiftung
